# 2079: Updates to the documentation system for R

**DOI:** 10.1017/cts.2017.20

**Published:** 2018-05-10

**Authors:** Andrew Middleton Redd

**Affiliations:** The University of Utah School of Medicine, Salt Lake City, UT, USA

## Abstract

OBJECTIVES/SPECIFIC AIMS: This research seeks to create a next generation documentation system that exists independent of but is complimentary to the packaging system in R. The new documentation can be manipulated programmatically as with all R objects. It also implements multiple translators for creating documentation from different sources, including documentation pages written in latex and code comments. METHODS/STUDY POPULATION: This work is based on input from the R Documentation Task Force, which is a working group, supported by the R Consortium and the University of Utah Center for Clinical and Translational Science, consisting of R Core developers, representatives from the R Consortium member companies and community developers with relevant interest in documentation. An abstraction of the documentation currently in use was created and extended. This abstraction was translated to a class system in R so that documentation can be stored and manipulated in R. RESULTS/ANTICIPATED RESULTS: The class system representing the documentation and the tools for creating the translators are currently being implemented in R. A preview of the system is scheduled to be available at the time of the conference. DISCUSSION/SIGNIFICANCE OF IMPACT: Good documentation is critical for researchers to disseminate computational research methods, either internally or externally to their organization. This work will facilitate the creation of documentation by making documentation immediately accessible and promote documentation consumption through multiple outputs which can be implemented by developers.

